# Reamed Exchange Nailing in Nonunion of Tibial Shaft Fractures: A Review of the Current Evidence

**DOI:** 10.7759/cureus.9267

**Published:** 2020-07-19

**Authors:** Kavyansh Bhan, Anshika Tyagi, Tejasvi Kainth, Apurv Gupta, Muhammad Umar

**Affiliations:** 1 Trauma and Orthopaedics, Whipps Cross University Hospital, London, GBR; 2 Orthopaedic Surgery, Maulana Azad Medical College, New Delhi, IND; 3 Language Access and Internal Medicine, Winnipeg Regional Health Authority, Winnipeg, CAN; 4 Surgery, Maulana Azad Medical College, New Delhi, IND; 5 Internal Medicine, Jinnah Sindh Medical University, Karachi, PAK

**Keywords:** tibia non-union, tibia nail, bone grafts, non-union, tibial shaft fracture

## Abstract

One of the most dreaded complications of fracture management is a nonunion. Nonunions are usually difficult to manage and can be a source of significant mental, physical, and financial distress to the patient. The incidence of nonunion is dependent on multiple factors including degree of comminution, open versus closed, concomitant infection, and vascular status, and therefore the management of such nonunions continues to be an often debated topic. Currently, there is no clear consensus on the role of reamed exchange nailing for tibial shaft nonunions. While reamed exchange nailing for aseptic tibial shaft nonunions has shown promising results, with very high union rates, many surgeons prefer newer novel techniques such as plating along with osteoperiosteal decortication or the use of more conventional compressive plating with bone grafts. The aim of this article is to critically review and understand the available evidence base on reamed exchange nailing in nonunion of tibial shaft fractures and to explore the other options available and their indications.

## Introduction and background

One of the most dreaded complications of fracture management is a nonunion. Nonunions are usually difficult to manage and can be a source of significant mental, physical, and financial distress to the patient. It is a well-known fact that the process of fracture healing is reliant on a plethora of factors, including the degree of comminution, fracture pattern, vascularity of the area, stability of fracture, and presence of infection, and hence the management of long bone nonunions is a hotly debated topic. The incidence of nonunions in tibial shaft fractures can vary from 80% for Gustilo-Anderson type IIIB fractures to 16% for Gustilo-Anderson type II fractures [1]. The socioeconomic burden associated with such high nonunion rates is immense. Nonunions often require specialist limb reconstruction services along with repeated surgical interventions and prolonged hospital stays [2]. This can cost the National Health Service (NHS) of the United Kingdom anywhere between £7,000 and £79,000 per patient [3]. Khunda et al. established an average cost of £26,000 only for the direct costs of patient care, excluding costs of CT scans, ultrasounds, bone morphogenetic proteins, and outpatient antibiotic therapy [4].

Currently, there is no clear consensus on the role of reamed exchange nailing for tibial shaft nonunions. While many studies have shown excellent outcomes in patients following reamed exchange nailing for aseptic tibial shaft nonunions, with a reported union rate of 76% to 96% [5], many other studies suggest the use of newer novel techniques such as plating along with osteoperiosteal decortication or more conventional compressive plating with bone grafts [6].

The intention of the current article is to critically review and understand the available evidence base on reamed exchange nailing in nonunion of tibial shaft fractures. This study tries to scientifically analyze the concept of reamed exchange nailing and to identify the reasons for failure and success in order to aid the surgeon in making a reasoned decision.

History

Initially referred to as “secondary intramedullary nailing”, early uses of nail exchange can be traced back to 1972 when Olerud and Karlström reported a series of 13 patients who underwent nail exchange as a secondary procedure owing to either nonunion or failed fixations with plates for tibial fractures [7]. Surprisingly, all 13 patients showed complete osseous union, with almost 90% of the patients reporting “excellent” or “good” functional outcome [7]. This was followed by Christensen publishing a series of nine patients who were managed with reamed exchange nailing for nonunions of both the tibia and femur and reported a 100% union rate after nailing [8]. Exchange nailing as an alternative option for the management of nonunions was published extensively throughout the 1980s and 1990s [5]. Although the results were encouraging, most of these studies failed to distinguish patients who underwent exchange nailing following primary fixation with nails from the ones who underwent nailing following the failure of other types of fixation such as plates [9-11].

Concept of reamed exchange nailing

Hippocrates was among the first people to describe nonunion in 400 BC [12]. Multiple attempts were then made to classify nonunions based on their etiology. It was hypothesized as early as 1842 that callus formation does not take place as long as there is frequent movement and disturbance at the fracture site [13]. This “callus formation" has since become the most used indicator to classify nonunions. Nonunions can be traditionally classified into avascular and hypervascular types [14]. Avascular nonunion is mainly caused due to inadequate blood supply, thereby leading to a poor potential for the bone-forming cells to grow. Radiologically, avascular or atrophic nonunions show little to no callus formation at the fracture site [14]. Hypervascular or hypertrophic nonunions, on the other hand, are linked to poor immobilization in the presence of adequate blood supply and a good healing potential. X-rays in such conditions show abundant hypertrophic callus formation, indicating that the fracture site is biologically active but lacks appropriate stability [15].

The technique of exchange nailing involves the removal of previously implanted intramedullary nail followed by reaming and replacement with a larger diameter nail. Reamed exchange nailing activates periosteal vascular reaction through reaming while providing greater stability with a larger diameter nail.

The tibial diaphysis receives much of its blood supply through the endosteal vessels of the nutrient artery [16]. Reaming of the canal has dramatic acute effects on this blood supply, often destroying the endosteal circulation to levels almost half of prereaming levels. In response to this drop, there is an exponential increase in the periosteal blood flow owing to the intense hyperemic reaction generated after reaming [17]. The increase in periosteal blood flow promotes new bone formation, which, in turn, helps in the bone union. Hupel et al. suggested that the devascularized cortical bone after reaming contains numerous empty vascular channels that develop connection with the periosteal circulation after reaming, thereby resulting in increased deposition of osteoblasts and promoting new bone formation [18]. Other factors, such as growth factor activation, and a provision of reamed osteogenic debris to the site of nonunion may also contribute to healing of nonunions [19].

Introducing an intramedullary nail, with an increased diameter in reamed exchange nailing, allows for a greater area of the cortex to be in contact with the nail, i.e., it produces a tight fit [19]. This gives rise to higher stability, reduced cyclical loading, and a greater resistance to bending forces as the nail acts as an internal splint. Additionally, interlocking the nail substantially increases the resistance to shortening forces as well as deformation, thereby improving stability of the construct by manifolds.

## Review

Patients with tibial nonunion may often present with pain at the fracture site or a continuous pain while weight-bearing on the affected limb. Most of the patients with nonunion often fail to demonstrate any signs of progressive healing for at least three continuous months over a nine-month period since the index traumatic injury [20]. Indication for an exchange nailing is quite straightforward in such cases but can get complicated in patients presenting with mild or no symptoms clinically while showing pathognomonic signs of nonunion on radiographs. Decision-making for such patients should involve active input from the patient and their relatives throughout the process along with careful counselling of the patient and their family. The possibility of having the complication of a broken nail if surgical intervention is not carried out for such cases should be clearly explained and documented.

In hypertrophic nonunions, wherein stability of fracture is the foremost concern, exchange nailing has emerged as the treatment of choice over the past few decades [21]. The use of a thicker nail together with locking screws provides a biomechanically more stable construct [5]. On the other hand, atrophic nonunions have reported good outcomes with both dynamization and reamed exchange nailing, provided there is minimal bone loss. Factors such as segmental bone defect, presence of deformity, bone loss, and sepsis play an important role in deciding whether the patient will respond favorably to exchange nailing. Court-Brown et al. suggested that failure rates increase significantly in cases that present with less than 50% osseous contact between fracture fragments [22]. Thus, multiple factors need to be considered before offering reamed exchange nailing to patients with nonunion of the tibial diaphysis.

Technique

Following removal of index nail, the surgical technique of reamed exchange nailing involves reaming of the medullary canal by an additional minimum 2 mm of the preceding ream size [23]. The recommended size of the exchange nail is largely dependent on the initial undersizing of the index nail. Re-insertion of statically locked nails that are 1-4 mm larger than the extracted nails is the common practice. This may be explained by the fact that fractures fixed with nails occupying less than 70% of the cortex have a much higher rate of nonunion as compared to fractures fixed with nails occupying 90% or more of the cortex [23]. A larger nail also helps improve the strength of the construct [5]. There have been varying reports over the use of static or dynamic locking in exchange nail. Abadie et al. reported a mean union time of 7.3 months in patients who underwent statically locked exchange nailing, as opposed to 7.9 months in patients who had dynamically locked exchange nails [24]. On the other hand, some researchers argue that dynamically locked nails allow a gradual compression while weight-bearing, thereby promoting osseous growth and hence a theoretically earlier union at the fracture site [25].

The role of partial fibulectomy in exchange nailing is often disputed. While many authors advocate partial fibulectomy as a routine procedure in conjunction with exchange nailing based on the belief that an intact fibula causes distraction at the fracture site [26], others have recommended against partial fibulectomy unless the fracture is poorly reduced and requires manipulation before insertion of the exchange nail [22,25]. However, a biomechanical study conducted by Thomas et al. showed that the anteromedial and anterolateral surfaces of the tibia were always in tension and thus a partial fibulectomy might not prove to be helpful at all under any circumstance [27]. Due to these reasons, further research into the role and use of partial fibulectomy is required before any recommendations regarding its use can be made.

A point of concern while reaming is the heat generated by a drill bit, which can be a probable cause of nonunion by causing necrosis [5]. Multiple studies have suggested the use of a sharp drill bit, or continuous changing of the drill bit along with constant cooling of the bone by means of continuous irrigation with normal saline [28]. Constant cooling and changing of drill bits help in avoiding bone necrosis, implant loosening at later stages, and possible infections. Moreover, if preoperative radiographs depict extensive sclerosis at the nonunion site or are suggestive of a large bone defect, it is advisable to open up the fracture site and attempt removal of either the sclerotic bone or address the bone defect with the help of autogenous bone grafting to avoid difficulties with reaming at a later stage.

Clinical results

Reamed exchange nailing has proven to be quite effective in both avascular and hypervascular nonunions. In a retrospective study conducted at South Korea, union rates of 94% were reported for tibial diaphyseal nonunions treated with reamed exchange nailing [29]. The authors suggested that owing to the high union rates with exchange nailing, it can be recommended as the first line of treatment for all kinds of aseptic tibial diaphyseal nonunions. Another prospective study consisting of 188 patients demonstrated osseous healing through a single exchange nail in 182 out of 188 aseptic tibial diaphyseal nonunions [30]. A systematic review of seven randomized control trials by Lam et al. suggested that reamed intramedullary nailing is superior to nonreamed intramedullary nailing, as reamed nailing had a superior union rate with reduced chances of postoperative infection [31]. In a large cohort of 102 tibial diaphyseal nonunions managed over a period of 21 years, Tsang et al. reported a union rate of 63% with a single nail exchange [32]. This figure improved dramatically to 93% if cases that required up to five repeated exchange nails were included. One of the major factors for the failure of exchange nailing was the presence of concomitant infection. It was hence hypothesized to consider other fixation modalities such as the Ilizarov treatment in the presence of infections with extremely resistant organisms [32].

Exchange nailing in septic tibial nonunion

Multiple studies have shown an increased risk of postoperative infections in patients undergoing exchange nailing after having sustained an initial open fracture [32-33]. Templeman et al. advised caution in attempting reamed exchange nailing in patients whose initial injuries were a Gustilo-Anderson type IIIB or higher [33]. They stressed the higher probability of such nonunions continuing to go for nonunion despite exchange nailing owing to continued infection and advised approaching such nonunions with alternative techniques such as bone grafting and augmentation plating. The authors also recommended a routine screening for infections in all tibial diaphyseal nonunions, including those with minimal to no risk of infection [33].

Bhandari et al. reported three independent predictors for reoperation of a tibial shaft fracture treated with intramedullary nailing, out of which an open injury was deemed to be the most important and the only predictor which could be medically managed by the surgeon [34]. Patients with open fractures have a higher tendency to go into nonunion. Petrisor et al. reported infection rates of 62.5% in patients with open fractures who were treated with reamed intramedullary nailing [35]. They went on to treat 18 infected tibial diaphyseal nonunions with reamed exchange nailing and reported osseous union in only 7 out of 18 cases [35]. Oh et al. discussed the possibility of chronic osteomyelitis in the entirety of the medullary canal in infected nonunions with a nail in situ [29]. They suggested a novel approach in the management of such cases by performing a medullary debridement with reaming followed by insertion of antibiotic beads and a staged exchange nailing after three weeks. This protocol helped the authors achieve a 100% union rate without the need for any further procedures. However, such complex management is associated with its own costs to the health care system and takes a toll on the quality of life of the patient. Tsang et al. reported prolongation of union time by almost three months in patients with septic tibial nonunions and reported that such cohorts usually require another surgical procedure to attain union [32].

Alternative treatment modalities for tibial nonunion with a nail in situ

Despite recent advances in the understanding of physiology of fracture union, management of tibial nonunion continues to pose a challenge to the treating orthopadic surgeon. A myriad of treatment options have been developed over the past few decades, and we now have options available for both conservative and surgical management of tibial diaphyseal nonunions. Aseptic tibial diaphyseal nonunions with a nail in situ have been commonly managed efficiently with augmentation plating along with bone grafting. Robert Judet in 1963 proposed osteoperiosteal decortication as a possible treatment option for tibial nonunions [36]. He hypothesized that elevation of cortical chips attached to the periosteum at the nonunion site in combination with mechanical support led to union over a period of eight months in 99% of the cases. Numerous studies have also discussed the role of a local bone marrow injection to induce osteogenesis and potentially heal the nonunion at a faster rate [37]. Likewise, a few studies have advocated the use of a percutaneous platelet-rich plasma injection to the nonunion site in order to enhance the delivery of platelet-derived growth factor (PDGF), transforming growth factor-beta (TGF-b), vascular endothelial growth factor (VEGF), and other growth factors to induce osteogenesis at the site of injection [38]. A few authors have suggested the use of electromagnetic fields for long bone nounions [37]. These electromagnetic fields act by enhancing the piezoelectric activity of the bones, which helps in generating positive electric potential, thereby boosting osteoblastic activity. Few recent studies have attempted to treat tibial diaphyseal nonunions through low-frequency ultrasound signals with the aim of generating nano-movements at the fracture site [39]. Although union was seen in 88% of the cases treated with ultrasound signals, the level of evidence for efficacy is low. Many other exciting options including biologic implants such as platelet gel and recombinant bone morphogenetic protein implant are under investigation and hence require further research before they can be widely recommended.

## Conclusions

Reamed exchange nailing is a highly effective treatment modality for nonunions of the tibial shaft. The surgery has a short learning curve, minimal blood loss, low associated morbidity, and a short hospital stay. With the option of axial alignment correction, reamed exchange nailing has shown extremely good functional outcomes in patients. However, the current literature suggests that exchange nailing is prone to failure and complications in patients with septic nonunions and substantial bone loss. In such cases, other modalities such as bone grafting with plating might prove to be more beneficial as compared to exchange nailing. In conclusion, a careful patient selection in combination with early identification of risk factors for nonunion can greatly improve the outcomes and aid the surgeon in achieving the best outcome.
